# Table Slide Shoulder Flexion test for early assessment following proximal humeral fracture: development and reliability

**DOI:** 10.1186/s12891-025-09170-1

**Published:** 2025-10-21

**Authors:** Brit Jorun Fossan Liseth, Rolf Moe-Nilssen, Randi M. Hole, Merete A. Malt, Inger Haukenes

**Affiliations:** 1 Department of Global Public Health and Primary Care, University of Bergen, Bergen, Norway; 2 Department of Physiotherapy, Haukeland University Hospital, Bergen, Norway; 3 Department of Orthopedic Surgery, Haukeland University Hospital, Bergen, Norway; 4 Research Unit for General Practice, NORCE - Norwegian Research Centre, Bergen, Norway

**Keywords:** Reliability, Test-retest, Proximal humeral fracture, Range of motion, Early rehabilitation, Pain, Goniometric measurement, Orthopaedic assessment

## Abstract

**Background:**

Proximal humeral fractures are common in older people, often resulting in pain and reduced shoulder range of motion, which impacts daily activities. Although early exercises are recommended to improve function, reliable methods for assessing shoulder range of motion in the early post-fracture phase are lacking. This study aimed to develop a method for measuring shoulder flexion early after non-surgically treated proximal humeral fractures and to assess its test-retest reliability.

**Methods:**

The novel Table Slide Shoulder Flexion test was piloted on 17 individuals, among them physiotherapists providing feedback. Within-day test-retest reliability was assessed by a single tester using an electronic goniometer. Shoulder flexion was measured in 37 patients (mean age 62 years, 86% women) with non-surgically treated proximal humeral fractures, 1–9 weeks post-injury. The goniometer was placed both in high-thoracic and mid-thoracic positions for reliability comparisons. Intraclass correlation coefficients (ICC) and Minimal Detectable Change (MDC) were calculated.

**Results:**

The method was deemed unsuitable for two patients, and one dropped out. In the remaining 34, ICC (2,1) reached 0.932 for the high-thoracic goniometer position and 0.926 for the mid-thoracic position when performing two table slides, with both values increasing further when three repetitions were performed. High-thoracic measurements slightly outperformed mid-thoracic measurements, achieving a MDC of 19° at the third repetition, compared to 20° for the third mid-thoracic repetition. Measured high-thoracic shoulder flexion ranged from 58–178°.

**Conclusion:**

The novel Table Slide Shoulder Flexion test is a reliable tool for measuring shoulder flexion in the early phase after proximal humeral fractures. It may be useful for tracking individual changes over time and as an outcome measure in group comparisons research. Further studies are needed to investigate its intertester reliability, responsiveness, and validity.

**Supplementary Information:**

The online version contains supplementary material available at 10.1186/s12891-025-09170-1.

## Background

Proximal humeral fractures (PHF) are common, accounting for approximately 5% of all fractures in European countries [1–3] with women making up around 70% of those affected [4, 5]. The incidence increases with age [6] and the total number of fractures rises due to the ageing population [3, 7, 8]. These fractures cause pain [9, 10]and they often lead to permanently reduced shoulder range of motion (ROM) [11–13]. Impaired shoulder ROM can cause difficulties with activities of daily living such as reaching for something on a shelf, grooming hair, dressing, carrying objects or throwing a ball [9, 14, 15] potentially diminishing independence and social life [9].

Approximately 80% of patients with PHF are treated non-surgically [4–6]. These patients are recommended to start the shoulder exercises within two weeks after the fracture [11] since an early onset has been shown to be safe [16] and improve function [11, 14, 17–19]. The exercises aim to restore mobility and function [20]. There is a lack of consensus regarding the further content of rehabilitation for these patients [20, 21]. Neither individual exercises nor entire rehabilitation protocols have been compared [11, 22], and there is a significant heterogeneity in rehabilitation protocols [23].

Measuring ROM is a foundational component in evaluating the effectiveness of mobility exercises. ROM data may inform key aspects of exercise prescription, including the extent to which ROM should be challenged, repetition dosage, pain provocation thresholds, muscular activation, and tolerance for compensatory movements. It may also guide the selection of specific exercises, such as functional use of the hand in daily activities, pendulum exercises, table slides, or wall slides. However, despite the emphasis on early mobilization, shoulder ROM during this initial phase has not been systematically measured.

One limitation of validated measurement methods for shoulder flexion is the requirement to raise the arm against gravity while the spine is positioned either vertically (i.e., sitting or standing) or horizontally (i.e., supine) [24]. This requirement is consistent across both active and passive measurement methods [24]. Due to pain and traditional concern of secondary fracture displacement [16] movement against gravity is not recommended in the early phase after non-surgically treated PHF. Pendulum and table slide exercises are recommended by major Norwegian orthopedic trauma centers [25, 26]. However, to our knowledge, no reliable methods exist for assessing shoulder ROM with the trunk in an anteriorly inclined position.

Despite challenges associated with introducing new assessment methods, researchers in the orthopedic field have argued that novel methods should be developed for use during early rehabilitation, when traditional, functional tests are avoided due to the risk of placing excessive strain on affected structures [27].

Reliability is a prerequisite for other measurement properties, such as validity [28]. The aim of this study was (i) to design a novel test suitable for the early assessment of shoulder flexion following non-surgical treatment of PHF, and (ii) to examine the test-retest reliability and measurement error for this test.

## Methods

This study with repeated measures design followed the definition of reliability provided by COnsensus-based Standard for the selection of health-related Measurement INstruments (COSMIN) [29, 30]. The study was planned according to the COSMIN checklist for assessing the methodological quality of studies on measurement properties of health status measurement instruments [30] and the scoring system for this checklist [31]. Moreover, we followed guidelines for reporting reliability studies [32] and recommended reporting of results from studies with instrument development [33]. For the classification of PHF, we used the AO/OTA Fracture and Dislocation Classification Compendium [34] where type A describes simple PHF involving one tuberosity *or* the metaphysis (Neer 2-part fractures), type B describes PHF involving one tuberosity *and* the metaphysis (Neer 3-part fractures) and type C describes PHF involving the anatomical neck or multifragmentary fractures (Neer 4-part fractures).

### Test development

The Table Slide Shoulder Flexion test measures shoulder flexion as the patient slides their forearm forward on a table while successively bending the upper body forward. The test was developed by the first author of this study, based on 18 years of clinical experience in orthopedic physiotherapy. Initial attempts using three-dimensional motion analysis proved unsuccessful due to obscured sternum markers during forward bending, leading to the choice of an electronic goniometer. Test procedures were piloted on 17 individuals: 7 with healthy shoulders, 3 with impaired shoulder movement, and 7 with healthy shoulders who were experienced physiotherapists. Feedback was collected from the 7 physiotherapists, all of whom were employed at a university hospital and experienced in exercises for patients with acute injuries, to refine the test procedures.

### Reliability study

#### Selection and description of participants

We consecutively invited all eligible patients from the public Minor Injury Department in Bergen, Norway, to participate in the study. They were included at the time of diagnosis or at follow up visits.

Inclusion criteria were a non-surgically treated PHF within 1–9 weeks after trauma; age > 18 years; ability to walk three meters with a crutch or cane; and the ability to speak Norwegian or English. Exclusion criteria were diagnoses in which pain from another body part was expected to exceed shoulder pain when performing table slide (i.e. femur or pelvis fracture); brachial plexus injury; inability to rest the forearm on the table (i.e. olecranon fracture); elbow ROM less than 30–90° (i.e. contracture, casted elbow); strong elbow spasticity, rigidity or tremor; and difficulties following instructions.

Patients were instructed not to take any analgesics during the 60 min before testing. Efforts were made to enroll as many participants as possible within a given time frame of 9 weeks. According to the formula of Giraudeau and Mary [35] as presented by de Vet [28] the resulting number of 37 participants corresponds to a sample size required for an intraclass correlation coefficient (ICC) value of 0.80, with a 95% confidence interval of ± 0.1, assuming three repetitions.

#### Technical information

The electronic goniometer EasyAngle (Meloq AB, Stockholm), a handheld digital device equipped with a gravity-based position sensor known as the MicroElectroMechanical System Inertial Measurement Unit (MEMS-IMU), was used for measurements. This position sensor allows the goniometer to calculate angles in two dimensions relative to a defined calibration position. EasyAngle has demonstrated reliability in measuring various joint movements [36–38].

#### Test-retest

One physiotherapist (the first author) conducted a single within-day test–retest reliability assessment for each patient. Patients performed three table slides in both the test and retest to obtain two data sets for analysis. The rest interval between test and retest was 3–10 min, determined by the patient´s acceptance of refreshments and their readiness for repeated measurement.

The Table Slide Shoulder Flexion test was conducted in a standard physiotherapy treatment room, using an armless chair (seat height 46 cm) and a height-adjustable table. The table height was adjusted to ensure that, when the patient sat upright, the shoulder (in 0° of flexion, abduction, and rotation), elbow (at 90° flexion), and hand were aligned in the same sagittal plane, allowing the forearm to rest entirely on the table.

#### The Table Slide Shoulder Flexion test procedure

Two key markings were made on each patient’s back: Mid-thoracic marking was selected to represent the apex of the thoracic kyphosis, as the tangent to the spine at this point was assumed to most closely align with the midaxillary line used in traditional measurement methods [24]. Marking was performed with the patient standing upright, based on the assumption that standing would yield more reproducible spinal curvature than sitting, as the latter may introduce greater variability due to pelvic tilt. EasyAngle was positioned vertically and calibrated to 0°. It was then moved along the patient´s spine until reaching 0° again, indicating the apex of thoracic kyphosis, where a horizontal line was drawn. High-thoracic marking was selected due to its greater proximity to the glenohumeral joint. Following palpation, a horizontal line was drawn at the level of the medial end of the scapular spine - a well-defined anatomical landmark [39] that approximately aligns with the glenoid cavity. High-thoracic marking was performed with the patient seated with the hands resting in the lap. This position was chosen because patients with proximal humeral fractures may present with elbow extension deficits due to fracture-related swelling in the upper and lower arm, or contractures unrelated to the fracture. A freely hanging arm with limited elbow extension may induce compensatory anterior tilting of the scapula due to gravitational forces, thereby reducing the reproducibility of scapular positioning if the degree of available elbow extension varies.

The patients sat sideways at the table, with the contralateral arm resting in the lap, ensuring no crossed thighs or bilaterally forward-stretched feet. The seated position was chosen to provide a broad and stable support surface, allowing patients to begin from a relaxed posture, to promote whole-body relaxation, and thereby enabling them to challenge their shoulder mobility more effectively. Mandatory positions of extremities were implemented to restrict spinal rotation and posterior pelvic tilt. Patients defined their level of “acceptable shoulder pain” on a Visual Analogue Scale (VAS) [40] before sliding their arm on the table to that level. To minimize pain exacerbation, given that the full protocol included six repetitions across test and retest sessions, patients were encouraged to perform only one single test repetition.

Patients were assured that table sliding to their defined pain level would not dislocate the fracture and were informed that shoulder stiffness is a long-term problem after PHF. Our assertion that the fracture would remain uncompromised during the movement was based on the premise that pain provocation was self-regulated; patients were repeatedly encouraged to remain within their self-defined pain level. Clinical support was provided through observation of body language and subjective pain reporting. No specific attention was given to fracture type or biomechanical factors. 15–20 min were allocated for the described patient preparation, before measurements were obtained.

Each test repetition was accompanied by the same instruction, emphasizing consistent speaking tempo and intonation. In the end position, patients indicated which body part prevented further movement. If the limitation involved the shoulder or upper arm, we deemed it fracture-related; if any other body part posed the limitation, we considered the method invalid for that patient.

**Fig. 1:**
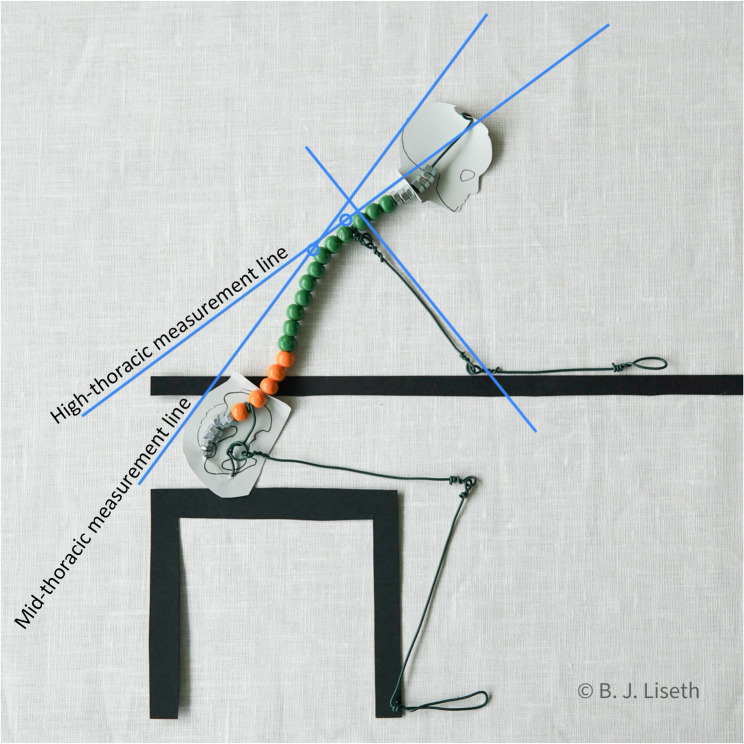
Measured shoulder flexion angles using thoracic reference points. High-thoracic and mid-thoracic markings served as origins for their respective measurement lines. The angles between these lines and humerus´ longitudinal axis were defined as high-thoracic and mid-thoracic measured shoulder flexion, respectively

In each end position, the humerus´ longitudinal axis was calibrated as 0° by aligning the goniometer from the lateral epicondyle to the pivot point of caput humeri [41]. High-thoracic registration involved reading the angle from the high-thoracic mark, while mid-thoracic registration followed a similar procedure with the mid-thoracic mark (Fig. 1). Marks on the back were removed and reapplied between test and retest, while table height remained unchanged. For a detailed procedure for the Table Slide Shoulder Flexion test, see Additional file 1.

#### Data analysis

Descriptive statistics were calculated. Homoscedasticity of the data was visually inspected through scatterplots between test and retest values.

To examine scoring consistency between test and retest, the intraclass correlation coefficient (ICC 2,1; two-way mixed model, absolute agreement, single measures) with a 95% confidence interval was used [28]. For average values, the ICC was calculated by first creating variables with the average values and then calculating the ICC between these. We used the definition of Koo & Li in which an ICC of less than 0.50 was considered poor correlation, 0.50–0.75 as moderate correlation, 0.75–0.90 as good correlation, and 0.90–1 as excellent correlation [42].

Within-subject standard deviation ($\:{\text{S}}_{\text{w}}$) was calculated as the square root of Within-People Total Mean Square [43] obtained from a two-way ANOVA table. Minimal Detectable Change (MDC) with 95% CI was calculated as $\:\sqrt{2}$ *1.96*$\:{\text{S}}_{\text{w}}$ [43]. IBM SPSS version 27 software was used for statistical analyses.

## Results

A total of 37 patients were included. Two patients stated that the belly prevented them from sliding the arm further and were not included in the study. One patient dropped out after retest 1. Except for missing values on retest 2 and 3 from this drop-out, all values from 35 patients were calculated.

Most patients were women (86%), mean age was 62 years (34–87 years) (Table 1) and none required walking aids. Data within fracture group A included fractures of collum chirurgicum and isolated fractures of tuberculum majus, with or without accompanying shoulder dislocation. No C fractures were included (Table 1).

**Table 1:** Sex, fracture laterality, age, time since injury and shoulder flexion in patients with PHF (*n* = 35 test 1, *n* = 34 test 2 and 3)

		All *n* = 35	A-fracture *n* = 18 (51%)	B-fracture *n* = 17 (49%)
Female	*n* (%)	30 (86%)	15 (83%)	15 (88%)
Right PHF Left PHF	*n* (%) *n* (%)	21 (60%) 14 (40%)	11 (61%) 7 (39%)	10 (59%) 7 (41%)
Age (years)	Mean (SD) Min-max	62 (11) 34–87	60 (12) 34–87	65 (9) 54–81
Time since injury (days)	Median (IQR) Min-max	20 (19) 10–62	22.5 (19) 10–50	13 (3) 10–62
High-thoracic measured shoulder flexion, mean (SD) from 3 tests and 3 retests (*n* = 34)	Mean (SD) Min-max	107° (33°) 58°–178°		
Mid-thoracic measured shoulder flexion, mean (SD) from 3 tests and 3 retests (*n* = 34)	Mean (SD) Min-max	92° (34°) 43°–166°		

*PHF* proximal humeral fracture, *A-fracture *simple PHF involving one tuberosity *or* the metaphysis, *B-fracture* PHF involving one tuberosity *and* the metaphysis, *SD* Standard Deviation, *IQR* Interquartile range

Measured high-thoracic shoulder flexion ranged from 58–178° (Table 1). High-thoracic measurements recorded values that were, on average, 15° higher ​​than mid-thoracic measurements (Table 1). In both measurements (high-thoracic and mid-thoracic) shoulder flexion increased by 2–3° from first to third repetition, as well as a 1° increase from test to retest. Test-retest difference was found to be homoscedastic.

In both measurements, the highest ICC was observed between the third test and its retest (Tables 2 and 3). High-thoracic measurements showed slightly superior results in all values compared to mid-thoracic measurements (Tables 2 and 3), with the highest ICC (0.958) obtained during the third repetition of table sliding (Table 2).

**Table 2:** Reliability for high-thoracic measured shoulder flexion (*n* = 35 test 1, *n* = 34 test 2 and 3)

Correlation between the two values of		Relative reliability	Absolute reliability			
		ICC (95% CI)	$\:{\text{S}}_{\text{w}}$	95% CI true value	MDC 95% CI	MDC 80% CI
Test 1	Retest 1	0.904 (0.818–0.950)	10.3°	± 20°	29°	19°
Test 2	Retest 2	0.932 (0.868–0.965)	8.8°	± 17°	24°	16°
Test 3	Retest 3	0.958 (0.918–0.979)	6.8°	± 13°	19°	12°
Mean of tests 1 and 2	Mean of retests 1 and 2	0.930 (0.864–0.964)	8.8°	± 17°	24°	16°
Mean of tests 2 and 3	Mean of retests 2 and 3	0.957 (0.916–0.978)	6.9°	± 14°	19°	13°
Mean of tests 1, 2 and 3	Mean of retests 1, 2 and 3	0.955 (0.912–0.977)	7.0°	± 14°	19°	13°

*ICC* Intraclass Correlation Coefficient, *CI* Confidence Interval, *S*_*W*_ Within-subject standard deviation, *MDC* Minimal Detectable Change

**Table 3:** Reliability for mid-thoracic measured shoulder flexion (*n* = 35 test 1, *n* = 34 test 2 and 3)

Correlation between the two values of		Relative reliability	Absolute reliability			
		ICC (95% CI)	$\:{\text{S}}_{\text{w}}$	95% CI true value	MDC 95% CI	MDC 80% CI
Test 1	Retest 1	0.887 (0.788–0.942)	11.6°	± 23°	32°	21°
Test 2	Retest 2	0.926 (0.858–0.962)	9.6°	± 19°	26°	17°
Test 3	Retest 3	0.957 (0.915–0.978)	7.2°	± 14°	20°	13°
Mean of tests 1 and 2	Mean of retests 1 of 2	0.917 (0.841–0.958)	9.8°	± 19°	27°	18°
Mean of tests 2 and 3	Mean of retests 2 and 3	0.954 (0.910–0.976)	7.45°	± 15°	21°	14°
Mean of tests 1, 2 and 3	Mean of retests 1, 2 and 3	0.947 (0.897–0.973)	7.8°	± 15°	22°	14°

*ICC* Intraclass Correlation Coefficient, *CI* Confidence Interval, *S*_*W*_ Within-subject standard deviation, *MDC* Minimal Detectable Change

$\:{\text{S}}_{\text{w}}\:$showed a similar pattern to the ICC, where high-thoracic measurements consistently outperformed mid-thoracic measurements (Tables 2 and 3) and the smallest (most favorable) $\:{\text{S}}_{\text{w}}\:$(6.8°) was obtained from the third repetition (Table 2). With this test methodology, a measured change of 19° or more indicates a 95% probability of a true change.

## Discussion

In a repeated-measures design, we examined test-retest reliability (ICC) of the novel Table Slide Shoulder Flexion test, designed for early assessment of shoulder flexion following non-surgical treatment of PHF. In this test, the patient slides the forearm forward on a table, while successively bending the torso forward. We found that this test had excellent test-retest reliability (ICC) when measured 1–9 weeks post-injury.

High-thoracic measured shoulder flexion demonstrated slightly higher reliability than mid-thoracic measurements, both in terms of ICC and $\:{\text{S}}_{\text{w}}$. Although minimal, the differences were consistently observed across all calculations. Anatomically, movement in the glenohumeral joint, which is expected to be reduced following a proximal humeral fracture, occurs closer to the high-thoracic segment than to the mid-thoracic. The glenoid fossa is located superiorly on the scapula, which itself rests primarily on the upper thoracic spine. It is possible that some patients reproduced similar shoulder flexion across tests and retest sessions, yet with varying degrees of simultaneous hip- and spinal flexion. We propose that the high-thoracic measurement was more sensitive to these variations, which may explain its slightly higher reliability.

Previous studies employing goniometers or inclinometers have reported ICC values between 0.80 and 0.99 for shoulder flexion in patients [44–50] and between 0.57 and 0.95 for asymptomatic individuals [41, 46, 50–52]. The findings from the present study are comparable to those with the highest ICC values, observed in patients who perform shoulder flexion actively while standing [48]passively while standing [45] and actively assisted in supine [47].

The smallest $\:{\text{S}}_{\text{w}}$ value achieved in this study (6.8°) was significantly larger than those in studies measuring shoulder flexion in young, asymptomatic individuals, which reported values as small as 2° [41, 46, 51]. It is suggested that those with a painful shoulder have greater movement variability than asymptomatic individuals [41]. This reasoning is supported by studies observing larger $\:{\text{S}}_{\text{w}}$ values in patients compared to asymptomatic individuals [46, 53] and in affected compared to unaffected shoulder in patients [44, 46, 47, 54, 55].

Terwee et al. [54] found lower absolute reliability in patients with severe pain or functional impairment compared to those with minor complaints and argued that pain and fear of overloading the shoulder were likely to explain the results. Direct comparison with their findings is limited, as Terwee et al. assessed shoulder movement in a direction other than flexion, and reliability metrics vary depending on the direction of movement being measured.

Our findings align with reported test-retest reliability values for goniometer- or inclinometer measured shoulder flexion in patient populations with severe pain. In patients with adhesive capsulitis, Tveitå et al. [49] reported an $\:{\text{M}\text{D}\text{C}}_{95}$ of 17° for passive shoulder flexion with scapular stabilization, and 28° for active shoulder flexion without scapular stabilization, both measured in a standing position. Similarly, in patients with subacromial impingement syndrome, Tozzo [44] reported $\:{\text{S}}_{\text{w}}$ values that, based on our calculations, correspond to $\:{\text{M}\text{D}\text{C}}_{95}$ values of 15° and 25° for passive shoulder flexion measured in seated and supine positions, respectively. Further, in patients with reflex sympathetic dystrophy, Geertzen [55] found an $\:{\text{M}\text{D}\text{C}}_{95}$ of 17° for active shoulder flexion measured in a standing position.

In a heterogenous group of patients with shoulder complains, Shin et al. [45] reported $\:{\text{S}}_{\text{w}}$ values that, based on our calculations, correspond to $\:{\text{M}\text{D}\text{C}}_{95}$ values ranging from 6° to 17°. A likely reason for their lower measurement error is that Shin et al. excluded individuals with less than 90° of active shoulder abduction against gravity, suggesting their population experienced less severe pain. Several studies have applied exclusion criteria such as high pain intensity [56], ROM under a certain level [45, 50] or inability to perform active shoulder flexion against gravity [46], which limits comparisons with patient groups exhibiting severe pain. Moreover, comparisons across studies are constrained because of the reporting of only ICC and not $\:{\text{S}}_{\text{w}}$ values [47, 48, 57–59]which are essential for calculating MDC.

Nonetheless, the large MDC values observed in populations with adhesive capsulitis [49], subacromial impingement syndrome [44] and reflex sympathetic dystrophy [55], as well as in our study´s population with proximal humeral fractures, highlight the challenges with measuring ROM in patients whose movement is limited by pain. These findings suggest that the primary source of measurement error stems from the patient populations themselves, rather than from the measurement technique.

Pain, being both sensory and emotional [60], complicates movement assessments. Any patient-controlled movement is influenced by fluctuating parameters like motivation, fear, and learning [49], as well as interaction between the patient and tester [55]. However, utilizing the patient´s self-defined pain threshold as ROM endpoint may offer advantages, as it is more likely to reflect the extent of extremity use in daily life compared to predetermined limits.

The discrepancy between the excellent ICC and yet large measurement error ($\:{\text{S}}_{\text{w}}$) is ascribed to sample heterogeneity, a factor strongly influencing ICC values [28, 61]. In the current study, patient inclusion varied in fracture severity and post-injury time, resulting in ROM heterogeneity. Similar discrepancies have been observed in other studies assessing shoulder ROM reliability in various patient populations [46, 55, 56]. This observation underscores that a larger measurement error, included a large MDC value, may be tolerated when assessing populations with large heterogeneity.

Increased patient involvement in defining and recording pain, along with prognostic information, was likely to enhance patient confidence in the table slide movement. This may have increased trust in sensory feedback, contributed to greater movement repeatability and thus enhanced the reliability of the measurement method.

### Strengths and limitations

A strength of this study is the consecutive enrollment of patients with PHF seeking health care in the Minor Injury Department in Bergen at several time points, minimizing selection bias. Therefore, the findings are applicable to patients seeking primary care and referred for non-surgical treatment, provided they do not meet the study’s exclusion criteria.

A second strength is the narrow confidence intervals surrounding the estimated parameters, demonstrating precision of these estimates. This suggests that, for early-assessed ROM in non-surgically treated proximal humeral fractures, the large MDC estimate is precise. Nonetheless, due to population heterogeneity, the Table Slide Shoulder Flexion test remains capable of differentiating among individuals, as supported by the high ICC.

Moreover, the test-retest design, unlike a strict intratester design such as measuring from a video recording, incorporates patient movement variability across repeated tests into the MDC. This aspect is crucial in clinical practice, where clinicians anticipate movement variability due to fluctuating pain experiences.

Reliability was assessed in conditions akin to clinical daily routines. This provides a realistic context for evaluating the test and makes the findings more likely to be directly applicable and relevant to healthcare professionals.

An additional strength is that an electronic goniometer is portable, lightweight, and reasonably priced (estimated cost of 300 euros), enabling widespread use. Alongside the goniometer, the Table Slide Shoulder Flexion test only requires an ordinary chair and a height-adjustable table, typically a physiotherapy treatment bench, which makes the test economically and practically accessible to numerous physiotherapists.

Patient demographics differed slightly from European epidemiological studies [1–5, 7, 62, 63] due to the recruitment only of non-surgically treated patients, from a publicly accessible clinic rather than an emergency department at a hospital, resulting in a younger age and higher female percentage. Nevertheless, the distribution of A and B fractures was like Swedish epidemiological data [62]. Reliability coefficients are population-specific [28, 64] and not necessarily transferable to all patients with PHF, nor to subgroups. Estimated reliability is inextricably linked to the investigated population. Further, our study did not examine whether the reliability of the measurement method is affected by fracture type, time since the injury, age, sex, pain or achieved ROM.

A limitation was the short time interval between test and retest. Retest interval decisions should not be based on convenience [65] but require careful consideration of the interval within which stability can be expected [66]. In our study, the short interval may have led patients to recall their technique, rather than reassessing pain tolerance as encouraged, potentially resulting in an overestimation of reliability. A longer time interval could have provided a more accurate representation of the true reliability of the test. However, we aimed to recruit as many patients as possible. Since several were recruited immediately after clinical treatment with a physiotherapist, we assumed that a less time-consuming study participation would increase recruitment rates. Additionally, limited clinical facilities necessitated a short interval. Most importantly, we chose a within-day test-retest design over a between-day design to avoid variations in movement due to fluctuations in pain levels across different days.

Some challenges in the design of the developed method should also be considered. Relinquishing control over the patients’ back movement enabled shoulder flexion without arm elevation, but this may simultaneously have decreased measurement reliability. Additionally, measurement errors may stem from back marker positioning, pressure on soft tissue during value reading and alignment of the line of sight along the length of the humerus. In our view, these last stated errors are clinically less significant than the impact of pain fluctuations on patient movement variability.

Similar to traditional methods for measuring shoulder flexion [24] this novel method quantifies flexion in the total shoulder complex, combining glenohumeral and scapular movements. Unlike traditional methods, this method demands good function across the movement apparatus during forward bending, particularly notable in individuals with extensive shoulder flexion, rendering it unsuitable for some. In our study, two of the PHF patients lacked validity in the measurement method, as both reported their belly touching their lap (both had a shoulder flexion more than 100°). This proportion warrants consideration when planning future applications of the measurement method.

We measured shoulder flexion using two reference points, mid-thoracic and high-thoracic, to investigate which yielded the most reliable results. In clinical practice, however, we do not anticipate that using both reference points provides additional value compared to one. Should future users of the test nonetheless opt to perform both measurements while streamlining administration, it is worth noting that no patients in our study exhibited an elbow extension deficit sufficient to visibly affect scapular positioning. This suggests that both measurement markings may be performed in a standing position.

## Conclusions

The Table Slide Shoulder Flexion test, developed for early assessment of shoulder flexion following PHF, effectively measures ROM before the fracture has fully healed. During the test, patients slide their forearm on a table while progressively bending their upper body forward until reaching a self-defined acceptable level of pain. This innovative test demonstrated excellent reliability among patients with non-surgically treated PHF, measured 1–9 weeks post-injury. The measurement error suggests that the test is suitable for tracking individual changes in shoulder flexion over time and can serve as a valuable outcome measure in studies involving group comparisons. Further research is warranted to investigate the intertester reliability, responsiveness, and validity of the Table Slide Shoulder Flexion test.

## Supplementary Information

Additional file 1. Procedure for the Table Slide Shoulder Flexion test. Detailed procedure, including positioning, instructions, and measurement technique.

## Data Availability

No datasets were generated or analysed during the current study.

## Abbreviations

PHF: Proximal humeral fracture
ROM: Range of motion
VAS: Visual Analogue Scale
ICC: Intraclass correlation coefficient
S_W_: Within-subject standard deviation
