# Supplementary material for: Table Slide Shoulder Flexion test for early assessment following proximal humeral fracture: development and reliability
Source: BMC Musculoskelet Disord. 2025 Oct 21;26:982. doi: 10.1186/s12891-025-09170-1 (PMC12538776; doi:10.1186/s12891-025-09170-1)
Supplement: Supplementary file 1 — Additional file 1. Procedure for the Table Slide Shoulder Flexion test. Detailed procedure, including positioning, instructions, and measurement technique. [file 12891_2025_9170_MOESM1_ESM.docx]

**Additional file 1. Procedure for the Table Slide Shoulder Flexion test**

## **Equipment**

- EasyAngle electronic goniometer
- Non-permanent skin marker
- Chair without armrests, standardized seat height (46 cm used in our study)
- Height adjustable table (physiotherapy treatment bench used in our study)

## **VAS scale introduction and pain definition**

Introduce the VAS scale:

- *This is a personal pain scale. This end of the line means 'no pain', and this end means 'worst imaginable pain.*

Define "acceptable shoulder pain":

- *Where is acceptable shoulder pain for you today?*

Supplementory instructions, depending on the patient's response:

- *When you have defined 'acceptable shoulder pain, I will ask you to slide your arm on the table until it is as painful as you have marked.*
- *'Worst imaginable pain' could be the worst pain you have experienced or imagined.*
- *'No pain' could be no pain at all or your chronic pain.*

## **Clothing**

- The affected arm and back of the patient must be visible. In our study, the patient was provided with a shirt and helped button it back-to-front. The sleeve was not put on the affected arm; instead, the shirt was guided under the armpit of the affected arm before being buttoned behind the back. Afterwords, any bra was unbuttoned to make the back visible.
- Remove any objects that might hinder forward bending (e.g. belt buckle, mobile phone in a pocket)

## **Mid-thoracic marking**

- Instruct: *Stand as normal and look straight ahead*, with arms hanging by the side.
- Calibrate EasyAngle, with no ruler connected to a vertical corner in the room. By this, vertical position is defined as 0°.
- Place EasyAngle on the patient's back, ruler still not connected. Orient the goniometer along the longitudinal direction of the spine so that the display is read from the lateral side. Ensure that the goniometer touches the dorsal spinoses and not the soft tissue.
- Move the instrument along the spine until it shows 0° (vertical position) (Figure 1), representing the apex of the thoracic kyphosis (around Th 6).
- Use a skin marker to draw a horizontal line at the height of the instrument’s center.


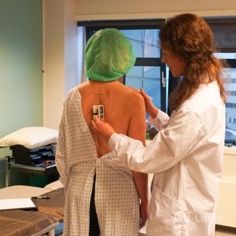


Figure 1: Mid-thoracic marking. The apex of the thoracic kyphosis is detected as the vertical position of EasyAngle. Previously performed, high-thoracic marking is visible.

## **High-thoracic marking**

Have the patient sit with buttocks all the way into the chair seat, no crossed thighs, and feet not stretched forward.

- Instruct: *Rest your hands in your lap so that your hands touch each other. Look straight ahead*. If the patient has a slumped position; correct this through manually placing your hand in the patients lumbopelvic area.
- Palpate spina scapula bilaterally for orientation (around Th 3).
- Use a skin marker to draw a horizontal line at the level of the medial end of the spina scapula on the side to be measured (Figure 2).


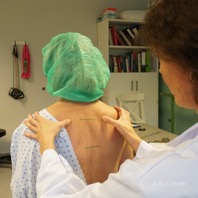


Figure 2: High-thoracic marking. Detect the height of the spina scapulae on the side to be measured through palpation. Previously performed, mid-thoracic marking is visible.

## **Chair and table setup**

- Place a standardized chair without armrests so the patient sits sideways at the table with the table edge touching their side.
- Start with the table lower than the elbow, so that the position of the upper arm is little affected when the hand is placed on the table. Ensure the upper arm is vertical and shoulder rotation is 0°. By this, ensure that shoulder, elbow and hand are in the same sagittal plane.
- Raise the table until the elbow rests on it. Take care that the table is not raised so much that the arm is raised; watch the elbow and the acromion. Adjust the table height for patient comfort if requested.

## **Prognostic information**

- Inform the patient that long-term stiffness is common after a proximal humeral fracture, and movement is necessary to regain mobility.
- Reassure that table sliding to acceptable pain provocation does not cause fracture displacement. In our study this claim was based on the fact that the patient controlled the pain provocation.
- Pay no attention to fracture type or biomechanical factors. Postulate no defined pain threshold. Provide support for the patient's body language and subjective assessment of the pain.

## **Test repetition**

- Instruct: *Slide your arm forward on the table, as far as you can, but no further than to an acceptable shoulder pain, as you have marked on the line*.
- Perform a visual instruction of the table slide as described in Table 1. Supplement with verbal instruction as described in Table 1 depending on the patient's response.
- Inform that the arm must rest in the end position while measurements are taken. Encourage the patient to consider this when choosing pain provocation. In our study several measures were collected, leading to a resting time of approximately 20 seconds in the end position.
- Encourage one single test repetition. Correct the movement if needed, according to descriptions in Table 1. If the patient pulls the arm back, apparently due to pain, suggest resting until the pain subsides. Suggest sliding the arm more slowly or not as far in subsequent measurements.
- The purpose of the test repetition is to ensure that the instructions are well understood, and the patient becomes familiar with pain provocation while resting in the end position. Allocate 15-20 minutes for VAS scale introduction, clothing, skin marking, prognostic information and test repetition.


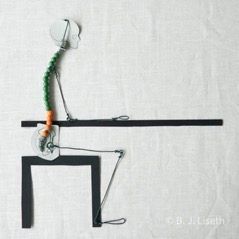

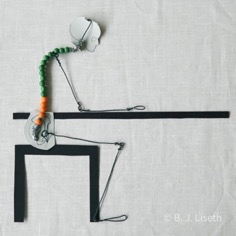

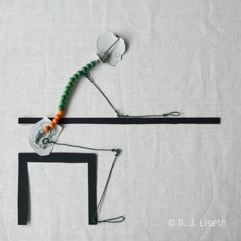


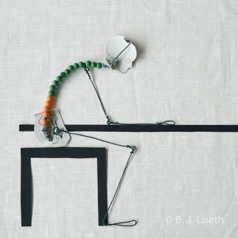

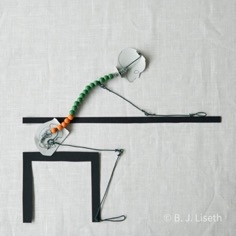

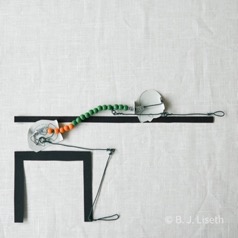


Figure 3. Some positions the patient could choose during table slide.

Table 1. Instructed, permitted and corrected sitting positions

|  | Injured arm | Buttocks | Feet | Unaffected arm | Trunk |
| --- | --- | --- | --- | --- | --- |
| Verbal instruction | *The elbow should touch the tabletop.*  *You may help the injured arm with the other hand.* |  | *Place your feet and other hand so that you can slide your injured arm as far forward along the table as possible, without experiencing more than acceptable pain.*  *Whatever feels best for you.* | | *You need to bend your upper body forward to slide your arm.*  *Look down when I measure.* |
| Visual instruction | Elbow touches tabletop.  Palm facing down.  Hand in the same saggital plane as elbow and shoulder. | Buttocks all the way into the chair seat. | Feet under knees. | Hand resting in lap. | No trunk rotation. |
| Patient choices that are permitted | Palm facing medially.  Active or passive shoulder movement (pull or push injured arm with opposite hand). | Sit at the front of the chair seat. | Crossed lower legs.  One foot stretched forward. | Support with hand or elbow on lap. |  |
| Patient  choices that must be corrected | If elbow is not touching tabletop.  Palm facing upwards.  Hand in different sagittal plane than elbow and shoulder. | Raising buttocks from chair seat. | Crossed thighs.  Both feet forward stretched. | Hanging arm. | Trunk rotation. Correct this manually and verbally with the words  *Look down when I measure.* |

**In the final position**, ask the patient: *What prevents you from sliding your arm further?* If the patient answers "shoulder", "upper arm", or touches any of these body parts, the movement restriction is considered associated with the fracture. If, despite attempts to adjust the sitting position, the patient indicates that a different body part restricts further movement, consider the measurement method unsuitable for the patient.

## **Measurement**

Repeat the instruction: *Slide your arm forward on the table, as far as you can, but no further than to an acceptable shoulder pain, as you have marked on the line.*

### Calibrate the longitudinal axis of the humerus as 0° (Figure 4)

1. Hook the ruler onto the goniometer.
2. Aim the ruler distally at the palpated lateral epicondyle of the humerus and proximally at the pivot point of caput humeri, located inferior to the posterior corner of the acromion (Kolber & Hanney, 2012).
3. Push the calibration button to define this position as 0°.
4. Remove the ruler.


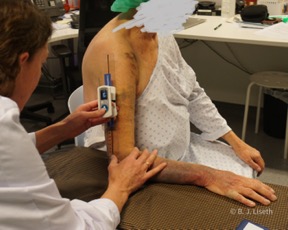
 A
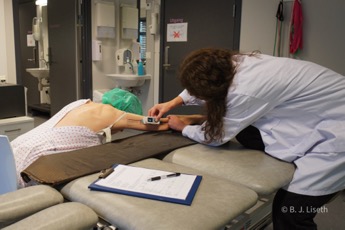
 B

Figure 4: Calibration of goniometer to 0° when aligned along the longitudinal axis of the humerus. Patient A sliding short and patient B sliding far.

### Perform high-thoracic measurement

1. Place the midpoint of the goniometer on the high-thoracic marking.
2. Orient the instrument along the longitudinal direction of the spine so that the display is read from the lateral side (Figure 5). Ensure that the goniometer touches the dorsal spinoses, not the soft tissue.
3. Read the angle value from the display.

In our study, this angle value was memorized, allowing the next measurement to be obtained without re-aligning the goniometer along the humerus.


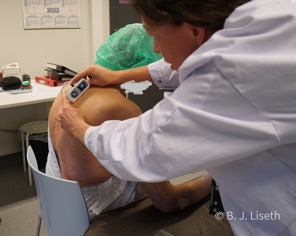
 A
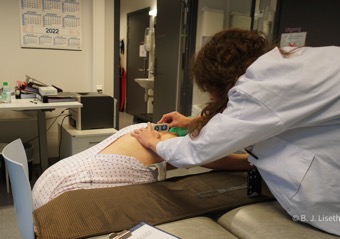
 B

Figure 5: High-thoracic measurement. Patient A sliding short and patient B sliding far.

### Perform midthoracic measurement

1. Place the midpoint of the goniometer on the mid-thoracic marking. Repeat the measures as described for high-thoracic measurement.

In our study, both memorized angle values were immediately noted in a table.


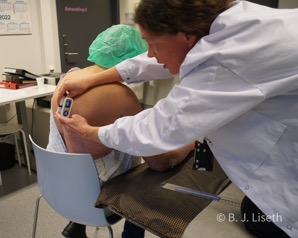
 A
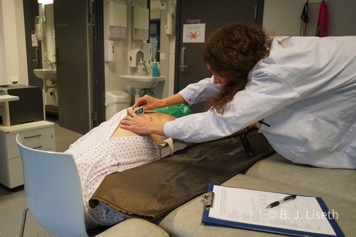
 B

Figure 6: Mid-thoracic measurement. Patient A sliding short and patient B sliding far.

## **For repeated measurements**

- Ensure the patient has the same level of analgesic effect during all measurements.
- Accompany each repetition of the table slide with the same instruction, maintaining consistent tempo and intonation.

**Pain registration**

After each table slide, when the arm is returned to a resting position, have the patient mark the pain experienced in end position on a VAS scale. This is done because the actual pain may differ from the planned, acceptable pain. For repeated measurements, use separate VAS scales.

**Clinical use**

It will be time efficient to record only one measurement: either high-thoracic or mid-thoracic. For repeated measurements, ensure that the same measurement is recorded every time. For questions, contact the corresponding author.
